# Characteristics of scrotal involvement in IgA vasculitis: Relationship with disease activity and inflammatory markers

**DOI:** 10.1007/s00431-025-06120-w

**Published:** 2025-04-10

**Authors:** Seyma Ertem, Zahide Ekici Tekin, Merve Cansu Polat, Didem Öztürk, Emine Özçelik, Mehveş Işıklar Ekici, Yasemin Uğur Es, Sultan Nilay Yoğun, Şeyma Erdem Torun, Cüneyt Karagöl, Banu Çelikel Acar

**Affiliations:** Division of Pediatric Rheumatology, Department of Pediatrics, University of Health Sciences, Ankara Bilkent City Hospital, 06800-Bilkent Ankara, Turkey

**Keywords:** Inflammation, IgA vasculitis, Pediatric vasculitis activity score, Scrotal involvement

## Abstract

Immunoglobulin A (IgA) vasculitis is a common systemic vasculitis in children, involving the skin, joint, gastrointestinal tract and kidneys. Scrotal involvement is a less common manifestation in the course of IgA vasculitis, which alters disease management. The purpose of this study was to present the characteristics of patients with IgA vasculitis with scrotal involvement and to compare patients with and without scrotal involvement. We also aimed to investigate the relationship between scrotal involvement and disease activity and inflammatory markers. This medical record review study was conducted in 234 male patients under the age of 18 years who were diagnosed with IgA vasculitis and followed for at least 6 months in the Pediatric Rheumatology Clinic. Demographic characteristics, clinical findings, laboratory findings, and pediatric vasculitis activity score (PVAS) of IgA vasculitis patients were recorded. CRP to albumin ratio (CAR), platelet-to-lymphocyte ratio (PLR) and neutrophil-to-lymphocyte ratio (NLR) were calculated using the patients’ complete blood count parameters and C reactive protein (CRP) levels. Two hundred thirty four male patients with IgA vasculitis were included in the study. Scrotum involvement was detected in 34 (14.5%) of patients. The mean age at diagnosis of 34 patients with scrotal involvement was 7.37 (4.41–8.43) years. Of the 34 patients, 15 had scrotal pain, swelling and rash, 12 had scrotal pain and swelling, and 2 had only scrotal swelling. Two (5.9%) patients had penile involvement with scrotal involvement. Patients were divided into two groups as those with scrotal involvement (*n* = 34, 10.2%) and those without (*n* = 200, 89.8%). Local edema, widespread skin involvement, hematuria, penile involvement, PVAS and CAR were significantly higher in IgA patients with scrotal involvement than in those without (*p* < 0.001, *p* < 0.001, *p* = 0.019, *p* = 0.001, *p* < 0.001, and *p* = 0.004, respectively).

*Conclusion*: Widespread purpura, local edema, penile involvement and hematuria are more common in patients with scrotal involvement than those without. PVAS and some systemic inflammatory markers such as CAR may be helpful in predicting scrotal involvement.

**Table:** 

**What is known:**
• *IgA vasculitis is the most common type of vasculitis in childhood and scrotal involvement is very rare during the course of the disease.* • *PVAS is a scoring system used to measure the severity of childhood vasculitis.*
**What is new:**
• *PVAS might be promising surrogate tool for predicting scrotal involvement in patients with IgA vasculitis.*

• *IgA vasculitis is the most common type of vasculitis in childhood and scrotal involvement is very rare during the course of the disease.*

• *PVAS is a scoring system used to measure the severity of childhood vasculitis.*

## Introduction

Immunoglobulin A (IgA) vasculitis is the most common systemic vasculitis in childhood and generally affects the vessels of the skin, renal and gastrointestinal (GI) tract. Patients typically present with palpable purpura, abdominal pain, GI bleeding, joint pain and swelling, hematuria, proteinuria and subcutaneous local edema. It is 1.3 to 2 times more likely to be seen in boys and observed most frequently between the ages of 5 and 10 years [1–4].

Approximately 2% to 38% of patients with IgA vasculitis have scrotal involvement [5–7]. Acute scrotal findings may be the first presenting symptom of IgA vasculitis [6]. Clinical findings are usually unilateral scrotal pain, swelling, and tenderness [8]. Imaging abnormalities such as epididymitis-orchitis, epididymitis also indicate scrotal involvement [6]. Rarely, testicular torsion, spermatic cord thrombosis, hematoma, and scrotal abscess may also be observed in IgA vasculitis [9].

A number of markers of systemic inflammation have been investigated in vasculitis due to their widespread applicability, low cost and ability to predict patient outcomes [10, 11]. Neutrophil-to-lymphocyte ratio (NLR) and platelet-to-lymphocyte ratio (PLR), markers based on neutrophils and lymphocytes, reflecting inflammation and activation of the immune modulatory pathway, have been evaluated. NLR was found to be higher in IgA vasculitis compared to healthy controls, and significantly increased in patients with GI tract complications [10]. Although there is evidence that NLR and PLR have an important role in assessing the presence and/or severity of GI tract and renal involvement in IgA vasculitis, there are also studies that do not support this association [12].

The Pediatric Vasculitis Activity Score (PVAS) was developed to assess the disease progression and response to treatments in childhood vasculitis [13]. Studies on childhood vasculitis have shown that the PVAS is a reliable scoring tool for the assessment of the disease and potential treatments [14, 15].

So far, studies on IgA vasculitis have generally focused on GI tract and renal involvement. However, the importance of presenting scrotal involvement is clear, which can affect patients’ quality of life, treatment approaches and outcomes. The purpose of this study was to present the characteristics and course of patients with IgA vasculitis with scrotal involvement and to compare the characteristics of patients with and without scrotal involvement. We also aimed to investigate the relationship between scrotal involvement and PVAS and inflammatory markers.

## Material and methods

This medical records review study was conducted in 234 male patients aged < 18 years who were diagnosed with IgA vasculitis according to the European League Against Rheumatism/Paediatric Rheumatology International Trials Organization/Paediatric Rheumatology European Society (EULAR/PRINTO/PRES) classification and followed up for at least 6 months between 2019 and 2024 in the Pediatric Rheumatology Department of our hospital [16]. Patients with diseases such as systemic disease, autoimmune disorder, malignancy that may cause vasculitis were not included in the study. Patients with a follow-up period of less than 6 months and missing data were also excluded.

### Data collection

Patients’ data were obtained from electronic medical records. Demographic characteristics, clinical findings, system involvement, laboratory findings at the time of diagnosis [white blood cell (WBC) count, lymphocyte and neutrophil counts, platelet count, C-reactive protein (CRP), and erythrocyte sedimentation rate (ESR)] were recorded. Imaging methods, treatment and prognosis of patients with scrotal involvement were noted.

### Definitions

Scrotal involvement was determined by the presence of scrotal edema, tenderness, erythema or abnormalities on scrotal color Doppler ultrasound (US). If only petechiae or purpuric rash was present in the scrotum, it was not considered as scrotal involvement.

Scrotal involvement was defined as thickening and swelling of the scrotal sac, epididymitis, orchitis, spermatic cord involvement, hydrocele, and increased testicular blood flow on color Doppler US. Testicular torsion was identified by twisting of the spermatic cord (whirlpool sign), redundant spermatic cord in the scrotal sac, heterogeneous hyper echogenicity, and mild to moderate thickening of the scrotal wall and diffuse reduction or absence of testicular blood flow on color Doppler US [5, 6].

The activity of IgA vasculitis was assessed using the Pediatric Vasculitis Activity Score. Nine organ based systems (general, mucosal and cutaneous membranes/ear/nose/throat, eyes, pulmonary, abdominal, cardiovascular, central nervous and renal systems) were assessed according to symptoms at disease onset. The presence or absence of each weighted item gave a maximum score for each organ system. The sum of the nine organ systems determined the total score representing each patient’s disease activity at the time of evaluation.

CAR, PLR and NLR were calculated using the patients’ complete blood count parameters and CRP levels at the time of diagnosis to predict disease severity. CAR was calculated as the CRP/albumin ratio. PLR was obtained by the ratio of platelet count to lymphocyte count. NLR was calculated by the ratio of neutrophil count to lymphocyte count.

#### Statistcal analysis

All statistical analyses were performed with SPSS ver. 16.0 (SPSS Inc., Chicago, IL, USA). Data with numerical variables were distributed according to the Kolmogorow-Smirnov test. All numerical variables fit the abnormal distribution. Therefore, median/IQR (25 th and 75 th percentile) values were calculated for all variables. In the comparison of numerical variables with/without scrotal involvement, Student -T test was applied for normally distributed numerical variables and Mann–Whitney-U for abnormal ones. Chi-square test was applied for categorical variables with or without scrotal involvement. We applied binary logistic regression analysis to identify independent predictors of scrotal involvement. In this study, when establishing for regression model, a limited number of outcome events can lead to uncertain and weak estimates. Additionally, the fundamental objective of regression analysis is to predict the dependent variable with the minimal number of independent variables. To mitigate this issue, the widely accepted rule of 10 events per variable was employed in this study. Factors associated with a p value < 0.1 in univariate analysis were further evaluated using multiple logistic regression analysis. The p value less than 0.05 was accepted for significancy. Receiver operating characteristic (ROC) curve analysis was performed to detect the cut-off value of the PVAS and CAR in predicting scrotal involvement with IgA vasculitis.

## Results

Electronic medical records of 266 male patients diagnosed with IgA vasculitis during the study period were reviewed. 17 patients with another disease causing vasculitis (1 systemic lupus erythematosus, 16 familial Mediterranean fever), 8 patients with a follow-up period of less than 6 months, and 7 patients with missing data were excluded in the study. None of the 32 patients excluded from the study had scrotal involvement. The remaining 234 male patients were included in the study.

The mean age at diagnosis of 234 patients was 8.00 (4.50–15.75) years. The mean follow-up period was 1.5 (1–3) years. At the time of diagnosis, all patients had skin involvement, GI tract involvement in 97 (41.5%), renal involvement in 20 (8.5%), arthritis and/or arthralgia in 77 (32.9%), and scrotal involvement in 34 (14.5%).

### Characteristics of IgA vasculitis diseases with scrotal involvement

Scrotal involvement was detected in 34 (14.5%) of male IgA vasculitis patients. The mean age at diagnosis of patients with scrotal involvement was 7.37 (4.41–8.43) years. Of the 34 patients, 15 had scrotal pain, swelling and rash, 12 had scrotal pain and swelling, and 2 had only scrotal swelling. Two (5.9%) patients had penile involvement with scrotal involvement.

Scrotal US revealed scrotal edema in 7 (38.9%) patients, scrotal fluid collection in 7 (38.9%) patients, scrotal wall thickening in 6 (33.3%) patients, epididymal swelling in 11 [61.1%; unilateral: 3 (16.7%), bilateral: 8 (44.4%)] patients, and increased testicular vascularity in 11 [61.1%; unilateral: 5 (27.8%), bilateral 6 (33.3%)] patients. CAR was 0.51 (0.15–1.07) in patients with unilateral and 0.37 (0.10–0.74) in patients with bilateral epididymal swelling (*p* = 0.620). PVAS was 2.5 (2.0–3.25) in patients with unilateral and 2.0 (1.0–3.0) in patients with bilateral epididymal swelling (*p* = 0.065).

Management of patients with scrotal involvement included supportive care (hydration, bed rest, analgesia and elevation of the scrotum) and steroid therapy. Of the 34 patients, 18 had only scrotal involvement, while 11 had scrotal involvement with GI tract involvement and 5 had scrotal involvement with proteinuria and/or hematuria. Those with only scrotal involvement received 1–2 mg/kg/day prednisolone. Steroid treatment was given for a median of 4 weeks. In 5 of 11 patients with scrotal involvement and GI tract involvement, 30 mg/kg/day pulse methylprednisolone was given for 3 consecutive days, then 2 mg/kg/day prednisolone was started. The remaining 6 patients were started on prednisolone at a dose of 2 mg/kg/day. The duration of steroid treatment was longer (median 6 weeks) considering GI tact involvement rather than scrotal involvement. Prednisolone was started to be tapered after 2 weeks in all patients. Although 2 patients with scrotal involvement had proteinuria, 3 had hematuria and one patient with both proteinuria and hematuria, these findings resolved in the follow-up and no change in treatment was required. Scrotal findings resolved within 1–3 days in all patients. Scrotal involvement did not recur in any patient.

### Comparison of IgA vasculitis patients with and without scrotal involvement

Patients were divided into two groups as those with scrotal involvement (*n* = 34, 14.5%) and those without (*n* = 200, 85.5%). Table 1 presents the characteristics of patients with and without scrotal involvement.

**Table 1:** Comparison of IgA vasculitis patients with and without scrotal involvement

	Patients with scrotal involvement (*n* = 34)	Patients without scrotal involvement (*n* = 200)	*p*
Age at diagnosis, mean (years, (min–max))	7.37 (4.41–8.43)	7.50 (5.79–10.39)	0.153
Clinical findings			
Skin involvement, n (%)	34 (100)	200 (100)	1
Lower extremities	23 (67.6%)	187 (93.5%)	** < 0.001**
Lower and upper extremities and trunk	11 (33.4%)	13 (6.5%)	** < 0.001**
GI tract involvement, n (%)	11(32.4%)	86 (43%)	0.244
Renal involvement, n (%)			
Hematuria	4 (11.8%)	6 (3%)	**0.019**
Proteinuria	3 (8.8%)	18 (9%)	0.973
Hypertension	0 (0%)	2 (1%)	0.558
Impaired renal functions	1 (2.9%)	19 (9.5%)	0.206
Joint involvement, n (%)			
Arthritis	12 (35.3%)	58 (29%)	0.459
Arthralgia	15 (44.1%)	62 (31%)	0.132
CNS involvement, n (%)	0 (0%)	1 (0.5%)	0.679
Local edema, n (%)	22 (64.7%)	26 (13.0%)	** < 0.001**
Foot	19 (86.3%)	23 (88.4%)	
Hand	2 (9.1%)	2 (7.6%)	
Forearm	1 (4.6%)	1 (5%)	
Penile involvement, n (%)	2 (5.9%)	0 (0%)	**0.001**
PVAS	2.0 (2.0–3.0)	2.0 (1.0–2.0)	** < 0.001**
Laboratory findings			
WBC (× 10^3^/μL, (min–max))	10.695 (9067–14927)	8855 (7095–11490)	**0.007**
Lymphocytes (× 10^3^/μL, (min–max))	3319.11 (940.00–12352.00)	2684.95 (920.00–7450.00)	0.126
Platelets (× 10^3^/μL, (min–max))	420.911 (319.250- 427.500)	362.910 (285.750–427.500	0.110
NLR (mean, min–max)	2.30 (1.27–4.20)	1.89 (1.32–3.58)	0.628
PLR (mean, min–max)	150.96 (104.41–200.29)	137.53 (101.56–183.15)	0.606
Hemoglobine (g/dL, (min–max))	12.13 (11.65–13.02)	12.25 (11.50–13.26)	0.756
CRP (mg/dL, (min–max))	6.0 (1.85–31.85)	3 (1.2–8.1)	0.006
ESR (mm/h, (min–max))	16. 0 (1.75–22.0)	10.0 (6.0–20.75)	0.187
Albumin (g/dL, (min–max))	44.0 (42.0–47.25)	44.0 (42.0–46.0)	0.763
CAR (mean, min–max)	0.15 (0.04–0.65)	0.07 (0.00–0.18)	**0.004**

GI: Gastrointestinal, CNS: Central nervous system, PVAS: Pediatric vasculitis activity score, WBC: White blood cell, NLR: Blood neutrophil-to-lymphocyte ratio, PLR: Blood platelet-to-lymphocyte ratio, CRP: C-reactive protein, ESR: Erythrocyte sedimentation rate, CAR: CRP-to-albumin ratio

There were significant differences in widespread skin involvement, local edema, hematuria, penile involvement in patients with scrotal involvement compared to those without (*p* < 0.001, *p* < 0.001and *p* = 0.019 and *p* = 0.001, respectively). PVAS was higher in patients with scrotal involvement compared to those without (*p* < 0.001). Similarly, there were significant differences in WBC and CAR in patients with scrotal involvement compared to those without (*p* = 0.007 and *p* = 0.004, respectively) (Table 1).

**Fig. 1:**
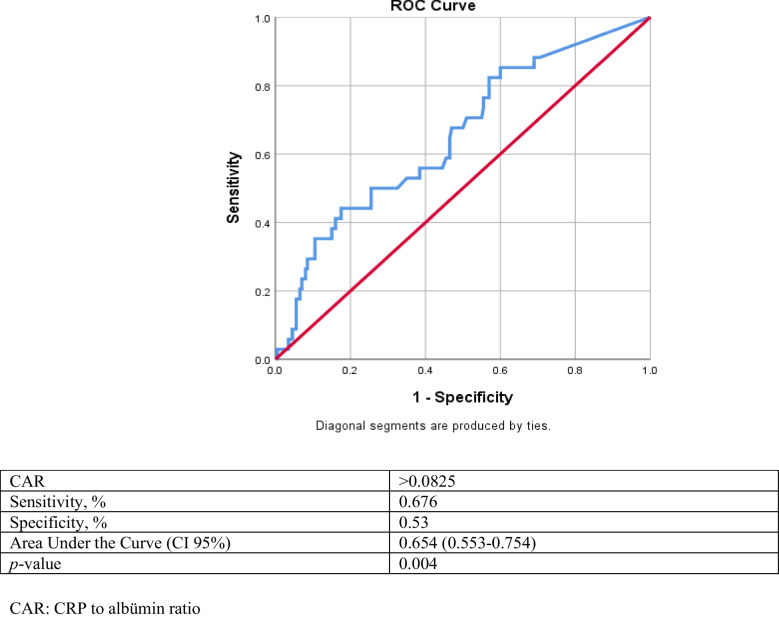
Receiver operating characteristic curve analyses conducted to determine the cut-off values for the sensitivity and specificity of CAR for predicting scrotal involvement

**Fig. 2:**
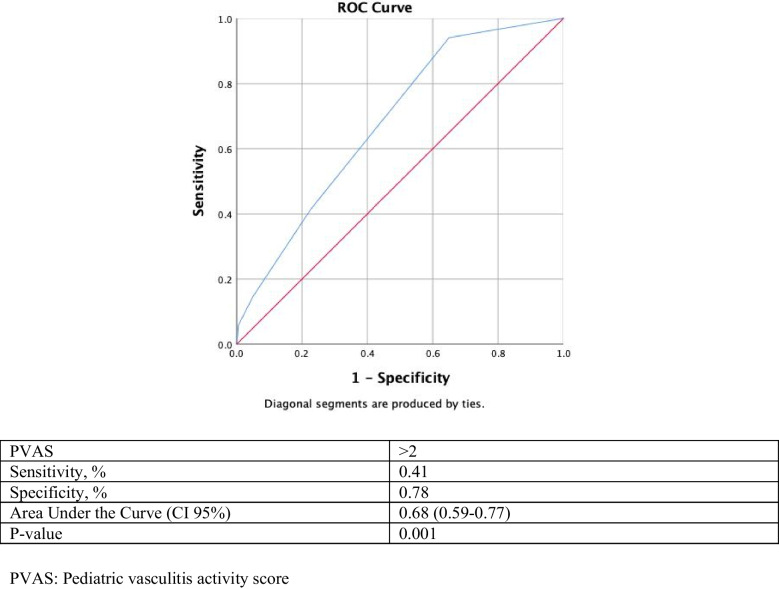
Receiver operating characteristic curve analyses conducted to determine the cut-off values for the sensitivity and specificity of PVAS for predicting scrotal involvement

Receiver operating characteristic analysis (ROC) of CAR and PVAS were performed to assess scrotal involvement. After analysis, CAR value > 0.0825 had a 67.6% sensitivity, 53% specificity, and the area under the curve (AUC) 65.4% (95% confidence interval 0.553–0.754) probability (accuracy) of predicting scrotal involvement (Fig. 1), and PVAS value > 2 had a 41% sensitivity, 78% specificity, and the area under the curve (AUC) 68% (95% confidence interval 0.59–0.77) probability (accuracy) of predicting scrotal involvement (Fig. 2).

After univariate and multivariate regression analysis, PVAS > 2 (odds ratio [OR] = 1.64; 95% confidence interval [CI]: 1.03–2.61, *p* = 0.038), widespread skin involvement (OR = 4.90; 95% CI: 1.64–4.65, *p* = 0.004), and local edema (OR = 8.17; 95% CI: 3.38–19.73, *p* < 0.001) were independently associated with scrotal involvement (Table 2).

**Table 2:** Independent predictor of scrotal involment by binary logistic regression analysis

Variables	Univariate			Multivariate
	OR (95%CI)	*p* Value	Adjusted OR (95%CI)	*p* Value
PVAS	1.94 (1.32–2.85)	**0.001**	1.64 (1.03–2.61)	**0.038**
CAR	1.62 (0.93–2.82)	0.088	1.61 (0.76–3.40)	0.210
Area of purpura	8.90 (3.65–21.71)	**< 0.001**	4.90 (1.64–14.65)	**0.004**
Local edema	12.27 (5.43–27.72)	**< 0.001**	8.17 (3.38–19.73)	**< 0.001**
Hematuria	4.31 (1.15–16.17)	**0.030**	2.26 (0.44–11.4)	0.326

CAR: CRP to albümin ratio, OR = Odds ratio, PVAS: Pediatric vasculitis activity score

## Discussion

The development of scrotal involvement in patients with IgA vasculitis is important as it may alter disease management. In the present study, scrotal involvement was shown to develop in approximately 14.5% of male patients with IgA vasculitis. Patients with scrotal involvement had more widespread skin involvement, local edema, penile involvement and hematuria than those without scrotal involvement. PVAS > 2 was found to be a predictor of scrotal involvement. However, an increase in systemic inflammatory markers such as NLR and PLR was not associated with scrotal involvement. On the other hand, CAR, the ratio of CRP to albumin, had higher specificity and sensitivity than the other two markers in predicting scrotal involvement.

Scrotal involvement develops rarely in patients with IgA vasculitis [5–7]. As found in our study, unilateral scrotum pain, tenderness and swelling are the most common clinical findings. While scrotal involvement is usually observed during the follow-up of the disease, it may occur as the first manifestation of the disease in only one fourth of patients [5, 6, 17, 18].

Imaging abnormalities such as enlargement of the epididymis, thickening of the scrotal skin, hydrocele with normal testicular blood flow may be detected [5, 19]. Scrotal US can both confirm scrotal involvement and reveal a critical complication such as testicular torsion. In our study, the most common US findings were epididymal swelling, increased testicular vascularity, scrotal fluid collection, scrotal edema, and scrotal wall thickening. Bilateral epididymal swelling and increased testicular vascularity were more common than unilateral. Fortunately, none of the patients had US findings of testicular torsion. However, it should be kept in mind that repeat US should be performed in patients with suspected testicular torsion during follow-up.

Given the need to adjust treatment in the presence of scrotal involvement, it is important to differentiate between patients with and without scrotal involvement. Thus, the clinician can closely follow patients who are likely to develop scrotal involvement. Ha et al. found that localized edema and central nervous system involvement were more common in patients with scrotal involvement [6]. Recently, it was shown that the frequency of scrotal involvement increased with renal involvement [8]. On the other hand, Buscatti et al. found no difference in terms of organ or system involvement in IgA vasculitis patients with or without scrotal involvement [5]. In our study, GI tract involvement, renal involvement and arthritis were similar in patients with and without scrotal involvement. However, widespread skin involvement and local edema were more frequent in patients with scrotal involvement compared to those without.

The PVAS, the pediatric version of the Birmingham Vasculitis Activity Score (BVAS), provides outcome scores for vasculitis. With PVAS, it may be possible to determine the activity and severity of vasculitis, provide a standardized management, and even predict some system involvement that may develop during the course of vasculitis. However, given the rarity of childhood vasculitis, there are very few studies presenting PVAS results in childhood vasculitis such as IgA vasculitis, Takayasu arteritis, and polyarteritis nodosa [20]. Feng et al. reported that PVAS is effective in assessing disease activity in Takayasu arteritis and its use is recommended in patients with renal damage [21]. Avci et al. showed that PVAS score was higher in IgA vasculitis with nephritis and also independent predictor for development of nephritis in patients with IgA vasculitis [22]. In previous studies, a threshold value of 2 was used to determine low and/or high disease activity in pediatric patients with vasculitis [15]. In our study, PVAS > 2 had 41% sensitivity, and 78% specificity for predicting scrotal involvement in patients with IgA vasculitis. PVAS > 2 also was independent predictor for scrotal involvement in patients with IgA vasculitis. Validation of the threshold value of the PVAS in larger scale studies and determination of different PVAS threshold values for the mild to severe spectrum of IgA vasculitis will enable more reliable and widespread use of this tool.

It was reported that markers such as NLR and PLR using the results obtained from complete blood count may reflect systemic inflammation and may even be prognostic indicators in some vasculitis [10–12]. Several studies have reported that NLR is a potential predictive marker of gastrointestinal bleeding in children with IgA vasculitis [23, 24]. The use of indices to predict renal involvement in IgA vasculitis has proven to be effective and it has even been emphasized that several indices can be appropriately combined to improve diagnostic efficiency [10–12]. It was found that these two markers may predict intravenous immunoglobulin resistance in Kawasaki disease. On the other hand, Liu et al. reported that these two markers were inadequate in Kawasaki disease [25]. Because of these contradictory results, the search for more useful markers such as CAR rate is ongoing in many diseases including vasculitis [26–28]. CRP, an acute phase protein, increases with systemic inflammation in vasculitis. Moreover, an expected decrease develops in albumin, which is considered a negative acute phase reactant. Changes in these two parameters result in a significant increase in the CAR. As a result, it is quite logical that CAR is more important than NLR and PLR. In our study, we investigated the predictability of CAR for scrotal involvement in patients with IgA vasculitis; and CAR > 0.0825 had 67.6% sensitivity, and 53% specificity after ROC analysis. However, in multivariate logistic regression analysis, CAR was not an independent predictor of scrotal involvement. Larger-scale studies are required for CAR for predicting important organ involvement.

The main approaches in the management of IgA vasculitis include supportive therapies such as hydration, bed rest and symptom relief [29]. Corticosteroids, azathioprine, cyclophosphamide and plasmapheresis are used in major organ or system involvement such as GI tract, renal or central nervous system [7, 18]. The severity of the disease, the affected organ or system and the presence or absence of life-threatening vasculitis determine the intensity of treatment. Ma et al. reviewed case reports published over a 35-year period and presented the treatment approach for 21 patients with IgA vasculitis with scrotal involvement. They reported that 16 patients received conservative treatment and steroids, 5 patients received only conservative treatment and 2 patients underwent surgical intervention. All patients had a good prognosis [18]. Treatment approaches for scrotal involvement vary from conservative to immunosuppressive therapy, and individualization of treatment is important. On the other hand, there are strong recommendations in support of steroid use. The European consensus-based recommendations for the diagnosis and treatment of IgA vasculitis in children-SHARE initiative recommend the use of steroids in orchitis [7]. In fact, steroid use has been reported in 93% of children with IgA vasculitis with scrotal involvement [5]. Ben-Chaim et al. reported relief of scrotal symptoms immediately after initiating steroid therapy [30]. In our center, patients with scrotal involvement were treated with a combination of conservative therapies and steroids.

The main limitations of this study are its retrospective design and single center. Due to the retrospective design of the study and the exclusion of patients with less than six months of follow-up and missing data, our results may reflect the findings of more severe or better documented cases. Prospective, multicenter studies may provide more accurate and generalizable results about the scrotal involvement of IgA vasculitis. In addition, the frequency of scrotal involvement in IgA vasculitis could not be given accurately since all patients did not have the same follow-up time. The lack of data on the use of PVAS in vasculitis is another limitation. On the other hand, the presentation of data on the use of PVAS in patients with scrotal involvement in IgA vasculitis is also a strength of this study. It is clear that there is a need for tools to measure the outcomes of vasculitis and in this present study, a cut off value was proposed for the predictive value of PVAS for scrotal involvement.

In conclusion, scrotal involvement may develop in IgA vasculitis and most patients are treated with steroids in addition to conservative therapies. Widespread purpura, local edema, penile involvement and hematuria are more common in patients with scrotal involvement than those without. PVAS and some systemic inflammatory markers may be helpful in predicting scrotal involvement.

## Data Availability

No datasets were generated or analysed during the current study.

## Abbreviations

IgA: Immunoglobulin A
CAR: CRP to albumin ratio
CRP: C reactive protein
ESR: Erythrocyte sedimentation rate
EULAR/PRINTO/PRES: The European League Against Rheumatism/Paediatric Rheumatology International Trials Organization/Paediatric Rheumatology European Society
NLR: Neutrophil-to-lymphocyte ratio
PLR: Platelet-to-lymphocyte ratio
PVAS: Pediatric vasculitis activity score
US: Ultrasound
WBC: White blood cell
